# Altered Capacity for H_2_S Production during the Spontaneous Differentiation of Caco-2 Cells to Colonocytes Due to Reciprocal Regulation of CBS and SELENBP1

**DOI:** 10.3390/antiox11101957

**Published:** 2022-09-30

**Authors:** Anne Sophie Scheller, Thilo Magnus Philipp, Lars-Oliver Klotz, Holger Steinbrenner

**Affiliations:** Institute of Nutritional Sciences, Nutrigenomics Section, Friedrich Schiller University Jena, D-07743 Jena, Germany

**Keywords:** SELENBP1, MTO, methanethiol, CBS, CTH, MPST, SQOR, H_2_S, differentiation, butyrate

## Abstract

Hydrogen sulfide (H_2_S) has been proposed to promote tumor growth. Elevated H_2_S levels have been detected in human colorectal cancer (CRC) biopsies, resulting from the selective upregulation of cystathionine β-synthase (CBS). In contrast, the recently identified novel H_2_S-generating enzyme, selenium-binding protein 1 (SELENBP1), is largely suppressed in tumors. Here, we provide the first comparative analysis of the four human H_2_S-producing enzymes and the key H_2_S-catabolizing enzyme, sulfide:quinone oxidoreductase (SQOR), in Caco-2 human colorectal adenocarcinoma cells. The gene expression pattern of proliferating Caco-2 cells parallels that of CRC, while confluent cells undergo spontaneous differentiation to a colonocyte-like phenotype. SELENBP1 and SQOR were strongly upregulated during spontaneous differentiation, whereas CBS was downregulated. Cystathionine γ-lyase and 3-mercaptopyruvate sulfurtransferase remained unaffected. Terminally differentiated cells showed an enhanced capacity to produce H_2_S from methanethiol and homocysteine. Differentiation induced by exposure to butyrate also resulted in the upregulation of SELENBP1, accompanied by increased SELENBP1 promoter activity. In contrast to spontaneous differentiation, however, butyrate did not cause downregulation of CBS. In summary, SELENBP1 and CBS are reciprocally regulated during the spontaneous differentiation of Caco-2 cells, thus paralleling their opposing regulation in CRC. Butyrate exposure, while imitating some aspects of spontaneous differentiation, does not elicit the same expression patterns of genes encoding H_2_S-modulating enzymes.

## 1. Introduction

Hydrogen sulfide (H_2_S) has a long history as an environmental toxin; however, it also serves as a gasotransmitter and an energy substrate in living organisms. The pleiotropic and in many cases bivalent (both stimulatory and inhibitory) actions of H_2_S do not only depend on its concentration and localization but also on the physiological and pathophysiological context. Biological effects of H_2_S are based on several mechanistic principles: (i) binding to and reactions on metalloproteins, (ii) direct interaction with reactive oxygen and nitrogen species (ROS/RNS), (iii) posttranslational modification of proteins through persulfidation of susceptible cysteine residues, and (iv) crosstalk with the two other gaso-transmitters, nitrogen monoxide (NO) and carbon monoxide (CO) [1,2,3].

**Figure 1:**
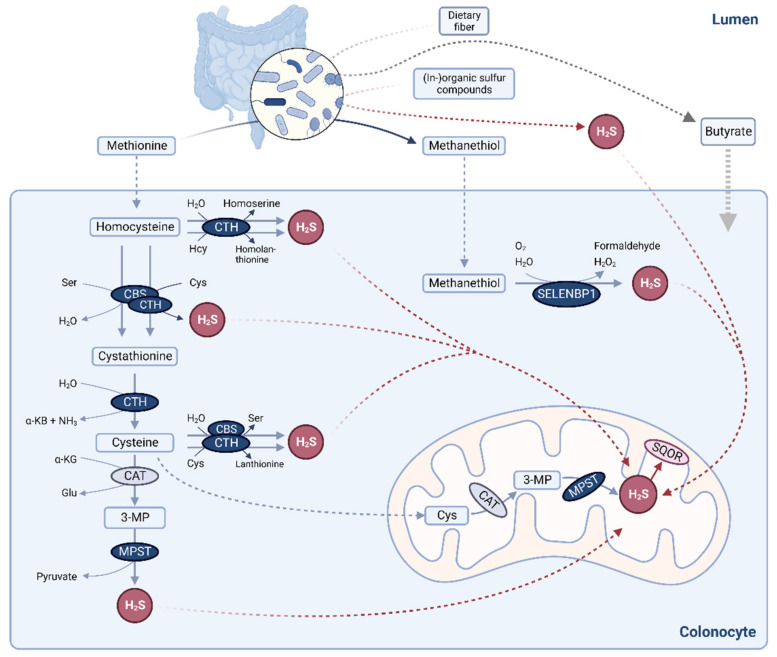
Overview of H_2_S biogenesis in the mammalian colon. Gut microbiota generate H_2_S by fermentation of dietary sulfur-organic compounds and by sulfate reduction (as well as butyrate by fermentation of dietary fiber). H_2_S production in colonic epithelial cells occurs through the four enzymes CBS, CTH, MPST and SELENBP1. Sulfur-containing amino acids serve as precursors for H_2_S production by CBS, CTH and MPST, while the SELENBP1 substrate methanethiol is produced from methionine by colonic microbiota. SQOR is a key enzyme in H_2_S catabolism. Scheme created with Biorender.com.

In humans, H_2_S has been detected in various tissues and also in serum [2]. Particularly high H_2_S production is found within the mammalian gut, due to the metabolic activity of commensal bacteria. Intestinal microbiota generate H_2_S through the fermentation of sulfur-organic dietary compounds, including cysteine, methionine and taurine as well as through reduction of sulfate [2,4] (Figure 1). Moreover, host cells, including enterocytes and colonocytes, contribute to H_2_S biogenesis in the gut [2]. Until recently, cystathionine β-synthase (CBS), cystathionine γ-lyase (CTH) and 3-mercaptopyruvate sulfurtransferase (MPST) were presumed to account for most of the endogenous H_2_S-producing capacity in mammals [5]. CBS and CTH, key enzymes of the transsulfuration pathway, catalyze a multitude of H_2_S-generating reactions, using cysteine and/or homocysteine as substrate(s) [5] (Figure 1). MPST may generate H_2_S from 3-mercaptopyruvate (3-MP), which derives from the cysteine aminotransferase (CAT)-mediated transamination of cysteine [5] (Figure 1).

In 2018, a novel human H_2_S-generating enzyme was identified by demonstrating the methanethiol oxidase (MTO) activity of selenium-binding protein 1 (SELENBP1) [6]. SELENBP1 catalyzes the conversion of methanethiol to H_2_S, hydrogen peroxide (H_2_O_2_) and formaldehyde [6,7] (Figure 1). High SELENBP1 levels were detected in the mature epithelial cells that are located at the top of the colonic crypts in close vicinity to the colonic lumen [7,8]. As methanethiol is primarily produced by intestinal microbiota, through methionine-γ-lyase (MGL)-catalyzed breakdown of methionine [9], this establishes another link between microbial and host sulfur metabolism. Methanethiol-derived H_2_S production within the colon has been known for more than 20 years [10]. Moreover, methanethiol has been detected in human flatus, at concentrations of 0.21 ± 0.04 µM [11].

Colonocytes are well adapted to cope with high levels of H_2_S, as they are capable of using it as an electron donor for ATP production in the respiratory chain [12]. H_2_S at concentrations of up to 20 µM stimulated oxygen consumption in colonic epithelial cells [13], whereas the mitochondrial respiration became inefficient when the supply of H_2_S exceeded its oxidation rate [12]. Catabolism of H_2_S occurs through the mitochondrial sulfide oxidation pathway, with sulfide:quinone oxidoreductase (SQOR) as the first and rate-limiting enzyme [14] (Figure 1).

Here, we compared the mRNA and protein levels of the four H_2_S-producing enzymes as well as the H_2_S-catabolizing enzyme SQOR and the capacity to produce H_2_S from different substrates in undifferentiated and terminally differentiated Caco-2 cells, a human colon adenocarcinoma cell line. The gene expression profile in proliferating Caco-2 cells was reported to parallel that in colorectal cancer (CRC) tissue, while their spontaneous differentiation upon contact inhibition mimics the development of absorptive polarized epithelial cells from stem cells in vivo [15,16,17]. Alternatively, Caco-2 cell differentiation can be induced by exposure to the short-chain fatty acid (SCFA) butyrate [18,19], a product of microbial fermentation of dietary fibers in the colon (Figure 1). Butyrate-induced differentiation occurs more rapidly than spontaneous differentiation, with shared characteristic features and some divergent patterns in the phenotype of the differentiated cells [20].

## 2. Materials and Methods

### 2.1. Culture and Differentiation of Caco-2 Cells

The Caco-2 cell line was obtained from the German Collection of Microorganisms and Cell Cultures (DSMZ, #ACC169; Braunschweig, Germany). Cells were cultivated in Minimum Essential Medium Eagle (MEM, #M4655; Sigma-Aldrich, Deisenhofen, Germany) supplemented with 20% (*v*/*v*) fetal bovine serum (FBS, #S0615; Sigma-Aldrich, Deisenhofen, Germany), 1% non-essential amino acids (#M7145; Sigma-Aldrich) and 1% penicillin/streptomycin (#P4333; Sigma-Aldrich) at 37 °C in a humidified atmosphere with 5 % (*v*/*v*) CO_2_ [7,21]. For spontaneous differentiation following contact inhibition [15,16], cells were grown until day 14 after reaching confluence. To that end, culture medium was replaced by medium containing 10% FBS on the day cells reached confluence, and renewed every 2–3 days thereafter until day 14 post-confluence (PC). For butyrate-induced differentiation [18,19], proliferating cells were treated with 1 or 2 mM sodium butyrate (#8175000100; Sigma-Aldrich) for 2 or 3 days. Intestinal alkaline phosphatase (ALPI) was used as differentiation marker, and its enzymatic activity was assessed spectrophotometrically in cell lysates [18,21] using para-nitrophenyl phosphate (#71768; Sigma-Aldrich) as substrate. Cell viability after butyrate treatment was assessed by Neutral Red assay, according to standard procedures.

### 2.2. Assessment of SELENBP1 Promoter Activity

Sequence information on the human SELENBP1 gene, which is located at chromosome 1q21-22 [22], was obtained from GenBank (accession number: AL391069). A 3,000 bp-genomic DNA fragment beginning immediately upstream of the translation start site of the predominant human SELENBP1 transcript variant 1 was amplified from genomic DNA by PCR, using Q5 DNA polymerase (New England Biolabs; Frankfurt am Main, Germany). Genomic DNA was isolated from Caco-2 cells with Monarch Genomic DNA Purification Kit (New England Biolabs). For PCR, we used primers introducing recognition sites for KpnI and HindIII restriction endonucleases, respectively: 5′-acgggtacctttatgccttacaggcatt-3′ and 5′-accaagcttgctgccgactggtacact-3′. The PCR product, after restriction digestion, was cloned into the firefly luciferase reporter gene vector pGL3basic (Promega; Madison, WI); the resulting plasmid was named hSELENBP1-luc.

For the reporter gene assays, Caco-2 cells were seeded in 6-well plates, and co-transfected at the following day with hSELENBP1-luc and pRL-SV40 renilla luciferase (Promega) plasmids using TurboFect transfection reagent (Thermo Fisher Scientific; Waltham, MA, USA). To determine background luciferase activity, the promoter/enhancer-less pGL3basic vector was co-transfected with pRL-SV40 in control experiments. At 48 h after transfection, cells were treated with sodium butyrate, and after another 48 h, luciferase activities were measured in a CLARIOstar plate reader (BMG LABTECH, Offenburg, Germany), using the Dual Luciferase Reporter Assay (Promega) [23].

### 2.3. RNA Isolation and Real-Time RT-PCR (qRT-PCR) Analysis

Total RNA was isolated using the RNeasy Mini Kit (Qiagen; Hilden, Germany), and converted into cDNA with RevertAid reverse transcriptase (Thermo Fisher Scientific). Thereafter, qPCR was performed in a CFX Connect cycler using SsoAdvanced Universal SYBR Green Supermix (Bio-Rad Laboratories; Munich, Germany) [24]. PCR amplicons were quantitated using CFX Connect software, version 3.1, and relative mRNA levels were computed after normalization to the reference gene, encoding 60S acidic ribosomal protein P0 (RPLP0) [25]. Primers were obtained from Thermo Fisher Scientific; their sequences are given in Table 1.

### 2.4. SDS-PAGE and Immunoblot Analysis

Lysates were generated by scraping the cells from dishes into HEPES-buffered saline (HBS; 50 mM HEPES, 150 mM NaCl, pH 7.4); these suspensions were lysed by freezing/thawing and sonication. Lysates underwent SDS-PAGE and were electroblotted onto PVDF membranes (Carl Roth; Karlsruhe, Germany) [7]. Equal loading and blotting of the proteins were confirmed by staining with 0.1 % Ponceau S solution in 5 % acetic acid. After blocking, membranes were probed with the respective primary antibody (Table 2), followed by incubation with suitable horseradish peroxidase (HRP)-coupled secondary antibodies (goat anti-rabbit IgG, #111-035-144, Dianova; Hamburg, Germany; or goat anti-mouse IgG, #31430, Thermo Fisher Scientific) and chemiluminescent substrate detection following standard procedures. Relative target protein levels were calculated after normalization to total protein loading in each lane on Ponceau S-stained membranes [26]. GAPDH was used as housekeeping protein and detected after stripping the previous antibodies off the membranes.

### 2.5. Determination of H_2_S Production in Caco-2 Cell Lysates

Cysteine (#1693.1; Carl Roth) and homocysteine (#H4628; Sigma-Aldrich) stock solutions were prepared in HBS. Equal volumes of lysates from differentiated (day 14) or undifferentiated (day 0) Caco-2 cells and of sulfur-containing amino acids were mixed. This resulted in reaction mixtures containing 1 mg/mL protein and either 50 mM homocysteine, 50 mM cysteine or 25 mM cysteine plus 25 mM homocysteine. Using suitable pH paper, no significant changes in pH of lysates were observed upon addition of amino acid solutions. Reactions were set up in a 384-well plate (Greiner Bio-One; Frickenhausen, Germany). Wells containing HBS and the above-mentioned substances, but no cell lysate, served as negative controls. All wells were covered with a 2% agarose gel containing 100 mM lead acetate, and incubated for 24 h at 37 °C. Thereafter, black PbS spots were detected on the agarose gel using a ChemiDoc^TM^ MP analyzer, and pixel density was determined using Image Lab^TM^ software, version 5.2.1 (Bio-Rad Laboratories). Measurement of methanethiol-derived H_2_S production occurred by a coupled MGL/MTO assay [7], with detection of the PbS spots on agarose gels, as mentioned above.

### 2.6. Statistical Analysis

Means were calculated from three independent experiments, and error bars represent standard deviations (SD). Statistical analysis was done using GraphPad PRISM software, version 8.0.1 (GraphPad Software; San Diego, CA, USA). Statistical analyses of the data obtained from qRT-PCRs and immunoblotting were done using the Friedman test and Dunn’s post-hoc test, while statistical significances of the data from the H_2_S measurements were calculated using Welch’s *t*-test due to heterogeneity of variances. The minimum level of significance was set to *p* < 0.05.

## 3. Results and Discussion

### 3.1. Alterations in mRNA Levels of H_2_S-Modulating Enzymes during Spontaneous Differentiation of Caco-2 Cells

H_2_S affects cell fate decisions such as proliferation, differentiation and apoptosis. Moreover, dysregulation of H_2_S homeostasis has been reported to promote cancer development and progression. In particular, altered expression of the H_2_S-generating enzymes CBS and SELENBP1 is associated with tumor degree and prognosis in many types of cancers including CRC [27,28]. While CBS, CTH and MPST have long been known as endogenous H_2_S sources in the intestine, SELENBP1 and H_2_S-catabolizing enzymes have been barely studied.

**Figure 2:**
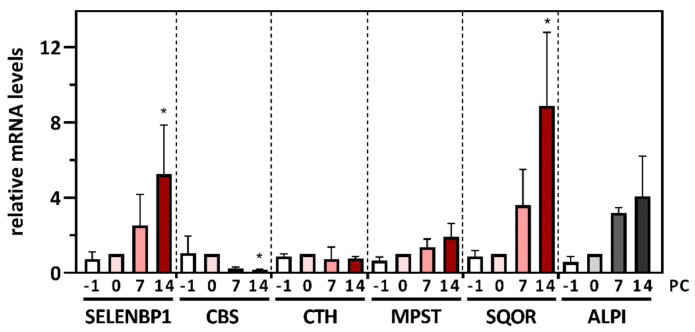
Alterations in the gene expression of H_2_S-modulating enzymes in the course of spontaneous (contact inhibition-induced) differentiation of Caco-2 cells to a colonocyte-like phenotype. Caco-2 cells were cultured for the indicated periods of time with respect to day 0, i.e., the day they reached confluence (PC, days post-confluence). Relative mRNA levels of SELENBP1, MPST, CBS, CTH and SQOR at different time points during spontaneous differentiation (as compared to the reference point PC 0) were determined by qRT-PCR, with normalization against the housekeeping gene RPLP0. Differentiation of the Caco-2 cells was substantiated by an increase in mRNA levels of ALPI. Three independent experiments were performed; the data represent means ± SD. Statistical analysis was done using the Friedman test and Dunn’s post-hoc test, with statistical significance at *p* < 0.05 (*).

To explore the regulation of all major human H_2_S-modulating enzymes, including SELENBP1 and the key H_2_S-catabolizing enzyme SQOR, in one in vitro model, we first analyzed alterations in their gene expression during spontaneous differentiation of the human intestinal cell line Caco-2 to a colonocyte-like phenotype. Relative mRNA levels were determined by qRT-PCR over the entire course of Caco-2 cell differentiation from proliferating to terminally differentiated cells, using the day of cell confluence that defines the beginning of contact inhibition-induced spontaneous differentiation as the reference point (day 0) for undifferentiated cells. The 60S acidic ribosomal protein P0 (RPLP0) was chosen as the housekeeping gene for normalization, as its gene expression has been reported to be particularly stable during the differentiation of human intestinal epithelial cells [25]. Gene expression of the digestive enzyme ALPI, whose biosynthesis and enzymatic activity is known to increase during Caco-2 cell differentiation [21,29], was elevated 4.0-fold (by trend; *p* = 0.11) in the terminally differentiated cells, as compared to undifferentiated cells (Figure 2), and was used as a marker of successful differentiation. The statistically significant (*p* < 0.05) upregulation of SELENBP1 and SQOR mRNA levels in the terminally differentiated cells was 5.2-fold and 8.9-fold, respectively. In sharp contrast, CBS mRNA levels were downregulated by 85% (*p* < 0.05) during differentiation. CTH and MPST mRNA levels remained largely stable (Figure 2). Moreover, the gene expression of CAT, which catalyzes the production of the MPST substrate mercaptopyruvate, did not differ between undifferentiated and differentiated Caco-2 cells (Figure S1).

The changes of SELENBP1, SQOR as well as CBS, induced by spontaneous differentiation were also detected at the protein level (see below), reflecting and confirming these changes in mRNA levels.

The observed differentiation-induced upregulation of SELENBP1 and SQOR is reminiscent of their localization in the human colon in vivo, where both enzymes are primarily found in mature epithelial cells located at the top of the crypts [8,13]. Moreover, the capacity to oxidize H_2_S has been shown to be higher in differentiated than in proliferating epithelial cells of the colonic mucosa [30]. Physiologically, the epithelial cells at the apical surface of the colonic crypts have to cope with the large amounts of H_2_S and methanethiol produced by commensal gut bacteria (Figure 1). Apart from its role as an enzyme in sulfur biochemistry, SELENBP1 appears to directly affect cellular proliferation: overexpression of SELENBP1 resulted in suppressed proliferation, whereas siRNA-mediated knock-down of SELENBP1 promoted proliferation [31].

The opposing regulation of CBS and SELENBP1 during Caco-2 cell differentiation is in accordance with previous reports regarding their role in carcinogenesis. A 7.5-fold increase in CBS protein levels was detected in samples of the CRC tissues of patients, as compared to the corresponding normal mucosa, whereas no remarkable changes in CTH and MPST levels were noticed [32]. Moreover, CBS was upregulated 6- to 12-fold in several CRC-derived cell lines, in comparison to a non-malignant colon cell line [32]. Immunohistochemical and immunoblotting analyses revealed a correlation of CBS expression with the degree of dysplasia in colonic epithelia [33]. The high CBS levels in tumor tissues from CRC patients resulted in the excessive production of CBS-derived H_2_S, which promoted carcinogenesis [33]. In contrast to CBS, SELENBP1 is downregulated in CRC, and the extent of SELENBP1 suppression inversely correlates with the prognosis for the patients [8,27,31]. In CRC tumor tissues of patients, a 3.5-fold decrease in SELENBP1 mRNA levels was observed, compared to normal mucosa [8]. Low SELENBP1 mRNA and protein levels were associated with cellular de-differentiation in CRC tissues [34]. Recently, the silencing of SELENBP1 has been reported to increase proliferation, migration and invasion of tumor cells, as well as the in vivo tumorigenesis of CRC [31]. Interestingly, elevated methanethiol concentrations were measured in the flatus from CRC patients, as compared to healthy individuals [35]. Methanethiol, which was proposed to be generated non-enzymatically in tumors through a Maillard reaction between glucose and methionine, was shown to promote cancer cell proliferation [35].

As mentioned before, SQOR was upregulated during spontaneous differentiation of the Caco-2 cells, in line with the primary occurrence of the sulfide oxidation pathway at the top of the crypts [13]. On the other hand, the comparison of paired normal mucosa and CRC tissues of patients as well as an in vitro analysis of non-malignant colon and CRC-derived cell lines indicated an upregulation of SQOR during carcinogenesis [13]. The normal apical-to-crypt gradient of SQOR expression in the healthy colon is lost in CRC tissue, resulting in high SQOR levels across the entire epithelium [13]. Thus, overall SQOR expression increases during carcinogenesis. Moreover, the increased SQOR expression might be advantageous for tumor cells due to its capability to stimulate energy production by transferring electrons from H_2_S to the respiratory chain [12,14], thereby promoting cancer cell growth.

### 3.2. SELENBP1 Promoter Activity Is Stimulated in Caco-2 Cells Undergoing Butyrate-Induced Differentiation

While the induction of SELENBP1 has been reported to be associated with terminal differentiation of cells from different developmental origin such as adipocytes, enterocytes/colonocytes and erythrocytes [8,36,37], the impact of differentiation on SELENBP1 promoter activity has not been explored yet. Thus, we cloned a 3000 bp-SELENBP1 promoter fragment from human genomic DNA for use in reporter gene assays. As transient transfection that is commonly applied for those assays does not work well in experiments studying spontaneous Caco-2 cell differentiation due to the long incubation time of 14 days, we sought to accelerate differentiation by exposure of the proliferating cells to sodium butyrate. Butyrate is present at concentrations of up to 20 mM in the colonic lumen [20]; it serves as an energy source for normal colonocytes but inhibits the proliferation of colon cancer cells [38]. Induction of Caco-2 cell differentiation by butyrate treatment was previously demonstrated, using ALPI as a marker protein [18,19,20].

**Figure 3:**
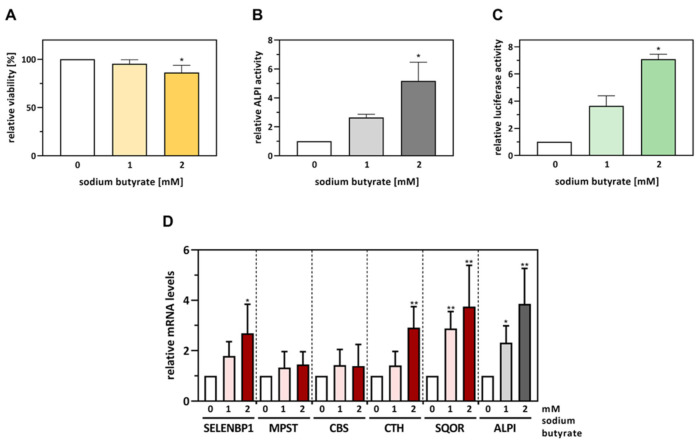
Butyrate increases SELENBP1 promoter activity and mRNA levels in parallel with the induction of differentiation in proliferating Caco-2 cells. The cells were cultured for one day and then incubated with sodium butyrate for 48 h at the indicated concentrations. (**A**) Cell viability was assessed by Neutral Red assay (untreated control set to 100%). (**B**) To monitor successful differentiation, enzymatic activity of ALPI was assessed photometrically in cell lysates. (**C**) Cells were co-transfected with hSELENBP1-luc and pRL-SV40 plasmids the day after seeding and then incubated with butyrate for another 48 h. SELENBP1 promoter activity was determined as relative luciferase activity (the untreated control was set as reference), with normalization against renilla luciferase activity. (**D**) Relative mRNA levels (with the untreated control set as the reference point) of the H_2_S-modulating enzymes and the differentiation marker ALPI in cells treated for 48 h with butyrate were determined as described in Figure 2. Three independent experiments were performed; the data represent means ± SD. Statistical analysis was done using the Friedman test and Dunn’s post-hoc test, with statistical significance at *p* < 0.05 (*) and *p* < 0.01 (**).

First, we checked for potential cytotoxicity of the applied doses of sodium butyrate by Neutral Red assay. Exposure to 1 mM butyrate for 48 h was not cytotoxic, while 2 mM butyrate provoked a slight but statistically significant decline in cell viability to 86% compared to untreated controls (Figure 3A), presumably due to the anti-proliferative and apoptosis-inducing effects of butyrate in proliferating Caco-2 cells [19]. Previous studies reported no cytotoxic effects when Caco-2 cells were exposed for 48 h to concentrations of up to 2 mM butyrate [18], as well as an induction of apoptotic cell death at a longer duration of the treatment [19].

Next, we examined the influence of butyrate on the differentiation state of the cells using the differentiation marker ALPI. ALPI activity was induced up to 5.2-fold (*p* < 0.05) by sodium butyrate in comparison to untreated Caco-2 cells (Figure 3B). The butyrate-induced increase in ALPI activity was associated with elevated SELENBP1 promoter activity: exposure to 1 mM and 2 mM butyrate stimulated SELENBP1 promoter activity 3.7-fold (by trend) and 7.3-fold (*p* < 0.05), respectively, as measured by the dual luciferase reporter assay (Figure 3C). While this points to a differentiation-mediated activation of the SELENBP1 promoter, the involved transcription factors as well as their binding elements on the promoter remain to be identified.

### 3.3. The Effects of Spontaneous Differentiation on mRNA and Protein Levels of H_2_S-Modulating Enzymes Are Only Partially Mimicked by Butyrate Exposure

As SELENBP1 promoter activity was increased upon exposure of Caco-2 cells to butyrate (Figure 3C), we next explored whether butyrate was capable of mimicking the effects of spontaneous differentiation on gene expression of SELENBP1 and the other H_2_S-modulating enzymes. In accordance with the stimulation of ALPI activity (Figure 3B), butyrate dose-dependently elevated ALPI mRNA levels in Caco-2 cells after 48 h of treatment, resulting in a 3.9-fold increased gene expression (*p* < 0.01) of ALPI by 2 mM butyrate (Figure 3D). In parallel, SELENBP1 and SQOR mRNA levels were elevated 2.7-fold and 3.8-fold, respectively, in Caco-2 cells treated with 2 mM butyrate (Figure 3D). Thus, butyrate upregulated gene expression of both the differentiation marker ALPI and two major H_2_S-modulating enzymes, SELENBP1 and SQOR; these effects of butyrate-induced differentiation were statistically significant but less pronounced compared to the outcome of spontaneous differentiation. Similar to spontaneous differentiation, MPST mRNA levels remained largely stable after butyrate exposure as well (Figure 3D). On the other hand, major differences between the two differentiation protocols emerged regarding the regulation of CBS and CTH. CBS mRNA levels were strongly suppressed during spontaneous differentiation, whereas butyrate treatment did not result in statistically significant changes in CBS mRNA expression. CTH mRNA levels were not affected during spontaneous differentiation but were elevated 2.9-fold (*p* < 0.01) by butyrate (Figure 3D).

**Figure 4:**
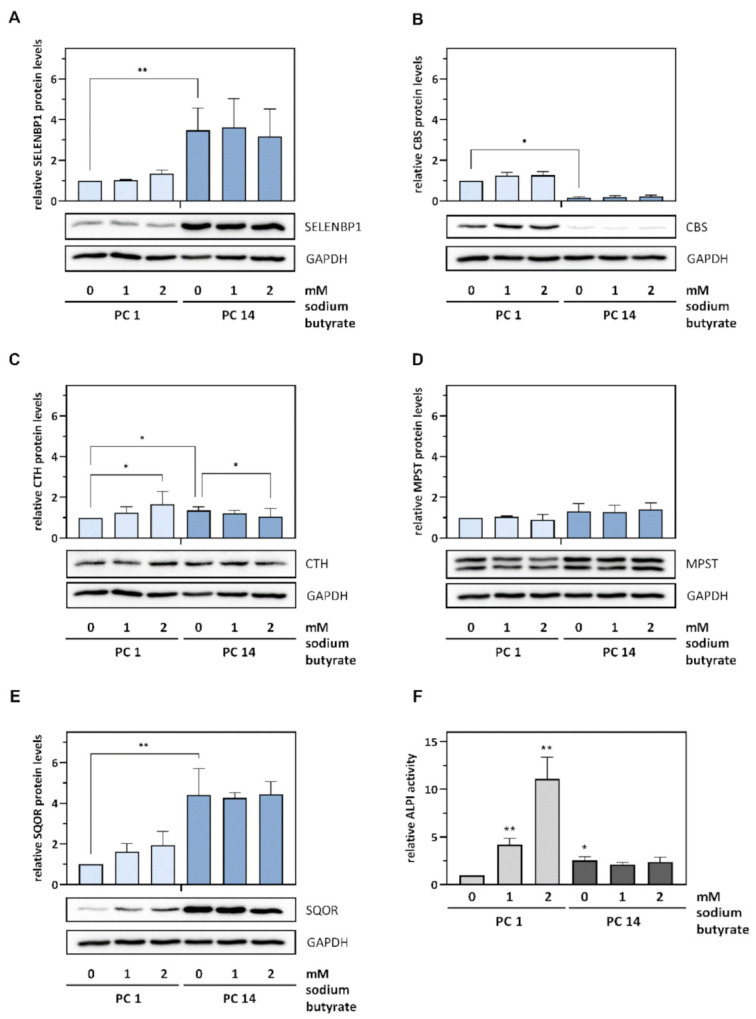
Spontaneous and butyrate-induced differentiation affect the protein levels of H_2_S-modulating enzymes in Caco-2 cells in a partially divergent manner. Proliferating and spontaneously differentiated cells were treated with sodium butyrate for 72 h until either day 1 (PC 1) or day 14 (PC 14) of differentiation. (**A**–**E**) Relative protein levels of SELENBP1, CBS, CTH, MPST and SQOR, respectively, in cell lysates were determined by immunoblotting and densitometric analysis of the blots, with normalization against Ponceau S-stained protein bands (the untreated control at PC 1 was set as reference). Three independent experiments were performed; the data represent means ± SD (upper panels). Representative immunoblots, with corresponding detection of the housekeeping protein GAPDH after membrane stripping, are shown (lower panels). (**F**) The relative enzymatic activity of ALPI in the lysates (the untreated control at PC 1 was set as reference) was measured photometrically to monitor differentiation. Statistical analysis was done using the Friedman test and Dunn’s post-hoc test, with statistical significance at *p* < 0.05 (*) and *p* < 0.01 (**).

Next, we compared the impact of butyrate treatment and spontaneous differentiation on the protein levels of the H_2_S-modulating enzymes in Caco-2 cells. The alterations in mRNA levels observed before were mirrored, for the most part, at the protein level, with distinct increases in SELENBP1 and SQOR expression and the suppression of CBS expression in the terminally differentiated cells that underwent spontaneous differentiation. When compared to spontaneous differentiation, the effects of butyrate-induced differentiation were less pronounced and did not reach statistical significance for the most part: Exposure of proliferating cells to 2 mM butyrate slightly increased SELENBP1 and SQOR protein levels 1.5-fold (by trend) and 2.0-fold (by trend), while spontaneous differentiation resulted in a 3.5-fold increase (*p* < 0.01) in SELENBP1 and a 4.4-fold increase (*p* < 0.01) in SQOR. MPST protein levels were barely affected and CTH was slightly but statistically significant upregulated by both differentiation protocols. As for CBS mRNA levels, CBS protein expression was differentially affected, with a strong and statistically significant decrease upon spontaneous differentiation but a slight and statistically not significant increase in cells undergoing butyrate-induced differentiation. Butyrate treatment of cells that were already terminally differentiated did not result in any alterations in protein levels of the H_2_S-modulating enzymes, with the exception of a slight decrease in CTH levels (Figure 4A–E and Figure S2). This was reflected by the different impacts of the butyrate treatment of proliferating and terminally differentiated cells on their ALPI activity, which was stimulated only if the proliferating cells were exposed to butyrate (Figure 4F). Moreover, ALPI activity was higher after spontaneous differentiation in the terminally differentiated cells, as compared to undifferentiated cells (Figure 4F).

Responses of Caco-2 cells to butyrate were previously reported to depend on the differentiation status: butyrate induced histone hyperacetylation, cell cycle arrest and ALPI activity in proliferating Caco-2 cells, whereas cells that underwent spontaneous differentiation before butyrate treatment were largely resistant [19]. Comparison between butyrate-induced and spontaneous differentiation revealed that both methods cause the stop of proliferation and an accumulation of cells in G0/G1 phase of the cell cycle. However, spontaneous differentiation resulted in broad concomitant upregulation of the characteristic brush-border enzymes, while butyrate selectively induced ALPI [20]. Those divergent phenotypic patterns may be due to the genetic reprogramming during spontaneous differentiation [16] that is probably more extensive than the changes in gene expression resulting from butyrate-induced histone hyperacetylation, and mimics the maturation of colonocytes during their migration from the base to the surface of the crypts in vivo [20].

### 3.4. Differentiation of Caco-2 Cells Alters Their Capacity for Substrate-Dependent H_2_S Generation

**Figure 5:**
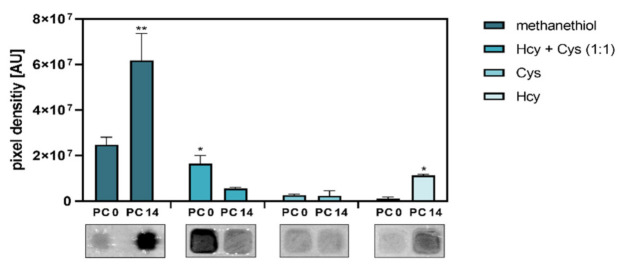
The capacity to produce H_2_S from methanethiol and cysteine analogues is differently affected by the spontaneous differentiation of Caco-2 cells, reflecting concomitant alterations of H_2_S-producing enzymes. Lysates of undifferentiated (PC 0) and terminally differentiated (PC 14) cells were incubated with substrates for H_2_S production (methanethiol, homocysteine (Hcy) and/or cysteine (Cys)) as indicated. Three independent experiments were performed, with one representative result shown for each substrate (lower panel). Pixel density of the obtained PbS spots was determined by densitometric analysis; the data represent means ± SD (upper panel). Statistical significances were calculated using Welch’s *t*-test, with *p* < 0.05 (*) and *p* < 0.01 (**).

In order to explore whether the pronounced alterations in the levels of H_2_S-producing enzymes upon spontaneous differentiation may affect the substrate-specific biosynthesis of H_2_S, lysates from undifferentiated and terminally differentiated Caco-2 cells were supplemented with methanethiol, cysteine (Cys) and/or homocysteine (Hcy) that serve as substrates for SELENBP1, CBS and/or CTH, respectively. Both methanethiol- and homocysteine-derived H_2_S biosynthesis were significantly elevated in the differentiated cells, whereas H_2_S production from cysteine was very low and unaffected by spontaneous differentiation (Figure 5 and Figure S3). It should be noted, however, that the lead acetate assay applied here is less sensitive for H_2_S detection than fluorescent probes [1]. We tested different Hcy and Cys concentrations, ranging from 250 µM to 50 mM, and found that a minimum of 25 mM was required to demonstrate H_2_S production by our assay (Figure S4). Due to this technical limitation, our data show the capacity of undifferentiated and differentiated Caco-2 cells to produce H_2_S from Cys and Hcy rather than their H_2_S production under physiological substrate conditions.

In contrast to the individual amino acids, incubation with a 1:1 mixture of homocysteine and cysteine resulted in substantially higher H_2_S release in the lysates of undifferentiated as compared to differentiated Caco-2 cells (Figure 5 and Figure S3). Similarly, undifferentiated Caco-2 cells showed a higher H_2_S production, as compared to differentiated cells, when Hcy/Cys mixtures with different ratios (1:2, 1:10, 1:25) were used (Figure S5).

The strong increase in methanethiol-derived H_2_S synthesis in the terminally differentiated cells reveals that the upregulation of SELENBP1 transmits to the level of enzymatic activity. Similarly, we have previously shown that SELENBP1 induction during differentiation of Caco-2 cells to a colonocyte-like phenotype comes with enhanced MTO activity [7]. Physiologically, the terminally differentiated colonocytes at the top of the crypts have to cope with and detoxify methanethiol that is produced from colonic microbiota.

To interpret our data on cysteine- and homocysteine-derived H_2_S production, one has to bear in mind that CBS and CTH are basically capable of processing both substrates (Figure 1) but with different preferences. CBS generates H_2_S predominantly through catalyzing a reaction with cysteine and homocysteine as co-substrates [39]. On the other hand, cysteine appears to be the preferred substrate for CTH, with 70% of CTH-catalyzed H_2_S production deriving from cysteine under physiological conditions [40]. However, if homocysteine levels are high, CTH can switch to generate H_2_S mostly from homocysteine, resulting in strong induction of H_2_S synthesis [40]. This CTH-mediated overproduction of H_2_S is also observed in CBS-deficient homocystinuria patients, who exhibit high plasma homocysteine levels, suggesting that CTH plays a major role in the detoxification of homocysteine in the absence of CBS [41], which otherwise would eliminate homocysteine through the transsulfuration pathway. Thus, our data suggest that H_2_S synthesis by CBS is diminished in terminally differentiated Caco-2 cells, while CTH-catalyzed H_2_S production is not affected by spontaneous differentiation.

## 4. Conclusions

**Figure 6:**
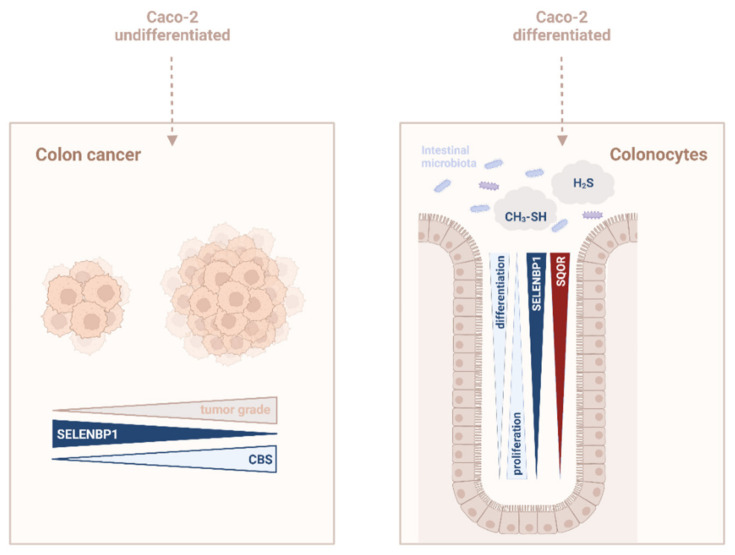
(Patho)physiological implications of changes in SELENBP1, CBS and SQOR levels in the course of CRC carcinogenesis and differentiation of intestinal epithelial cells. Left panel: The pattern of gene expression in undifferentiated Caco-2 cells parallels that of colon cancer. Elevated H_2_S levels, resulting from selective upregulation of CBS, have been found in human CRC biopsies, and were proposed to promote tumor growth. In contrast, SELENBP1 is largely suppressed in tumors. Right panel: Confluent Caco-2 cells cease proliferation and spontaneously differentiate to a colonocyte-like phenotype, associated with an increase in SELENBP1 and SQOR expression. High SELENBP1 and SQOR levels in mature colonocytes in vivo ensure the efficient catabolism of bacteria-derived methanethiol and H_2_S at the host–microbiome interface. Scheme created with Biorender.com.

SELENBP1 and CBS are reciprocally regulated during spontaneous differentiation of Caco-2 cells to a colonocyte-like phenotype, thus paralleling their opposing regulation in the tumor tissue of CRC patients (Figure 6). Nevertheless, it remains to be elucidated whether different enzymatic sources of H_2_S may affect the fate of epithelial cells at physiological substrate concentrations in vivo in a different way and whether this may affect the prognosis of cancer patients. SELENBP1 represents the H_2_S-producing enzyme whose upregulation was most pronounced during differentiation. Besides SELENBP1, SQOR, a key enzyme in H_2_S catabolism, was elevated in both spontaneously differentiated and butyrate-treated cells. Differentiation-induced upregulation of SELENBP1 and SQOR illustrates the need of the absorptive colonocytes that reside at the top of the crypts to cope with high amounts of methanethiol and H_2_S at the intestinal host–microbiome interface (Figure 6). Moreover, high SELENBP1 levels may affect cellular redox signaling, as two of the products of methanethiol oxidation, H_2_S and H_2_O_2_, act jointly to induce the persulfidation of susceptible cysteine residues in proteins; increased occurrence of this H_2_S-mediated posttranslational modification has been demonstrated in the presence of H_2_O_2_ [1].

Butyrate exposure, while imitating some aspects of spontaneous differentiation, does not elicit the same expression patterns of genes encoding H_2_S-generating enzymes, particularly regarding CBS expression.

## Figures and Tables

**Table 1:** Primers (5′–3′) used for qRT-PCR analyses.

Gene	Gene-ID	Forward Primer	Reverse Primer
ALPI	NM_001631	cctgccgttggaccttcac	acaaatctgcagtctctgggg
CAT	NM_002079	gctgaccggattctgaccat	cgttgattcgaccacttggc
CBS	NM_000071	gtttgagcggtgctgaactg	tatcctctggggaccccttc
CTH	NM_001902	ccagcactcgggttttgaat	gtgctgccactgctttttca
MPST	NM_021126	catggcggagccaggaag	gtagatcacgacgtgggtgg
RPLP0	NM_001002	acatgctcaacatctccccc	caggactcgtttgtacccgt
SELENBP1	NM_003944	cagcgcttctacaagaacga	tgatcaggcctggcattt
SQOR	NM_021199	gaagggagcgaaggtttttgc	gtctctcactgggctcaaca

**Table 2:** Primary antibodies used for immunoblot analyses.

Antibody	Supplier	Catalog Number
rabbit anti-CBS	Cell Signaling Technology	14,782
rabbit anti-CTH	Sigma-Aldrich	HPA023300
mouse anti-GAPDH	Sigma-Aldrich	G8795
rabbit anti-MPST	Sigma-Aldrich	HPA001240
mouse anti-SELENBP1	Medical & Biological Laboratories (Nagoya, Japan)	M061-3
rabbit anti-SQOR	Sigma-Aldrich	HPA017079

## Data Availability

Data is contained within the article and Supplementary Material.

## Supplementary Materials

The following supporting information can be downloaded at: https://www.mdpi.com/article/10.3390/antiox11101957/s1, Figure S1: Relative mRNA levels of cysteine aminotransferase (CAT) during the spontaneous differentiation of Caco-2 cells; Figure S2: Changes in protein levels of H_2_S-modulating enzymes in Caco-2 cells induced by spontaneous differentiation as compared to butyrate treatment (*n* = 3 experiments); Figure S3: Substrate-dependent H_2_S production in lysates from undifferentiated and terminally differentiated Caco-2 cells (*n* = 3 experiments); Figure S4: H_2_S production in lysates from undifferentiated and terminally differentiated Caco-2 cells, trying different concentrations of cysteine and homocysteine; Figure S5: H_2_S production in lysates from undifferentiated and terminally differentiated Caco-2 cells, trying different ratios of cysteine and homocysteine concentrations.
