# Supplementary material for: Altered Capacity for H2S Production during the Spontaneous Differentiation of Caco-2 Cells to Colonocytes Due to Reciprocal Regulation of CBS and SELENBP1
Source: Antioxidants (Basel). 2022 Sep 30;11(10):1957. doi: 10.3390/antiox11101957 (PMC9598602; doi:10.3390/antiox11101957)
Supplement: Supplementary file 1 [file antioxidants-11-01957-s001.zip › antioxidants-1899162-supplementary.pdf]

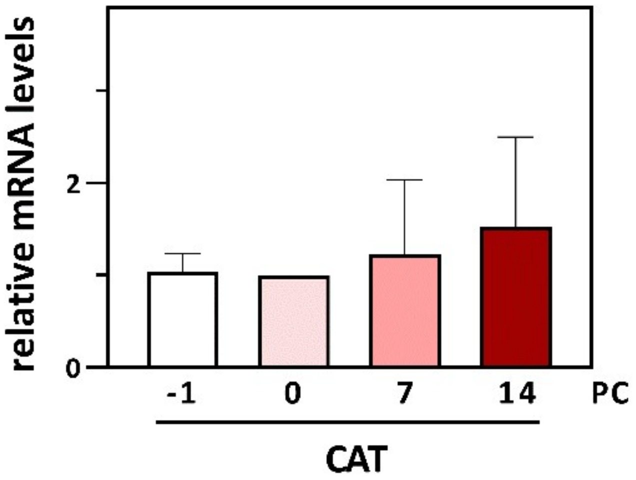

Figure S1. Relative mRNA levels of cysteine aminotransferase (CAT) during the spontaneous differentiation of Caco-2 cells.

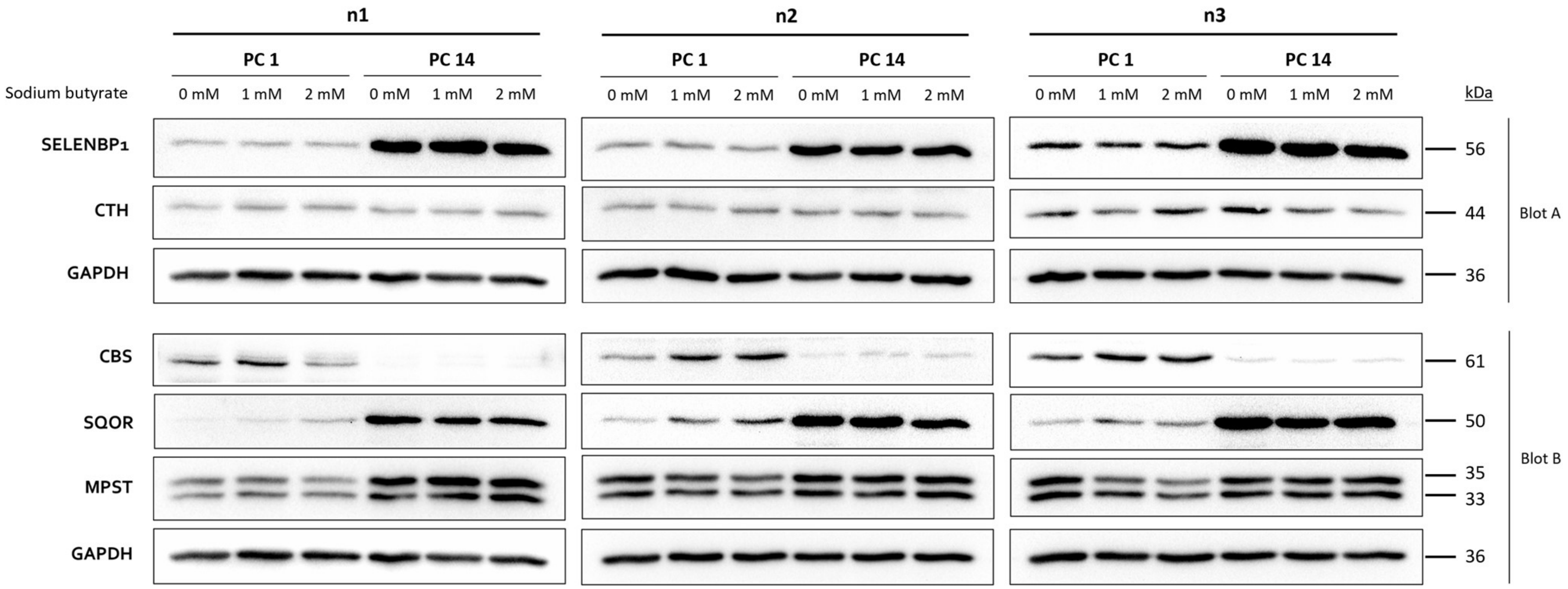

Figure S2. Changes in protein levels of H_2S-modulating enzymes in Caco-2 cells induced by spontaneous differentiation as compared to butyrate treatment (*n* = 3 experiments).

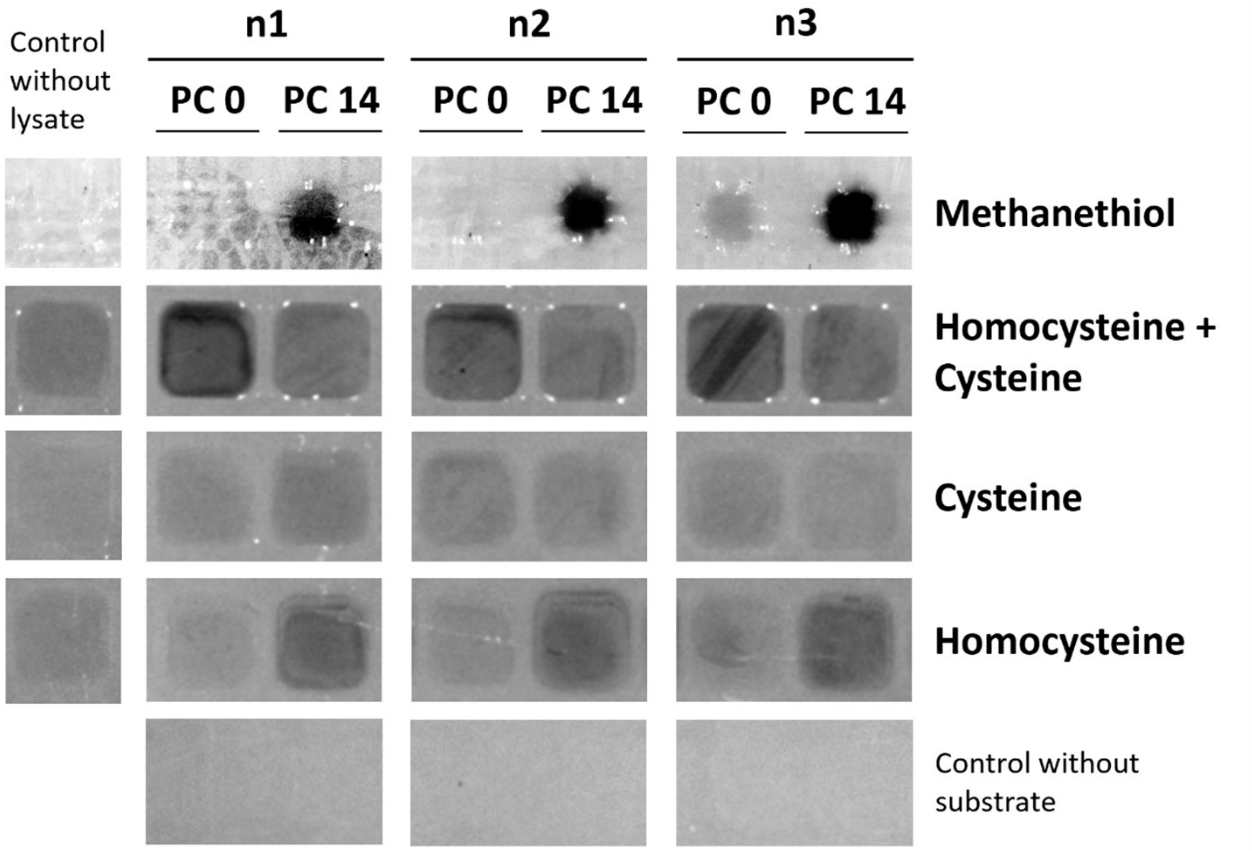

Figure S3. Substrate-dependent H_2S production in lysates from undifferentiated and terminally differentiated Caco-2 cells (*n* = 3 experiments).

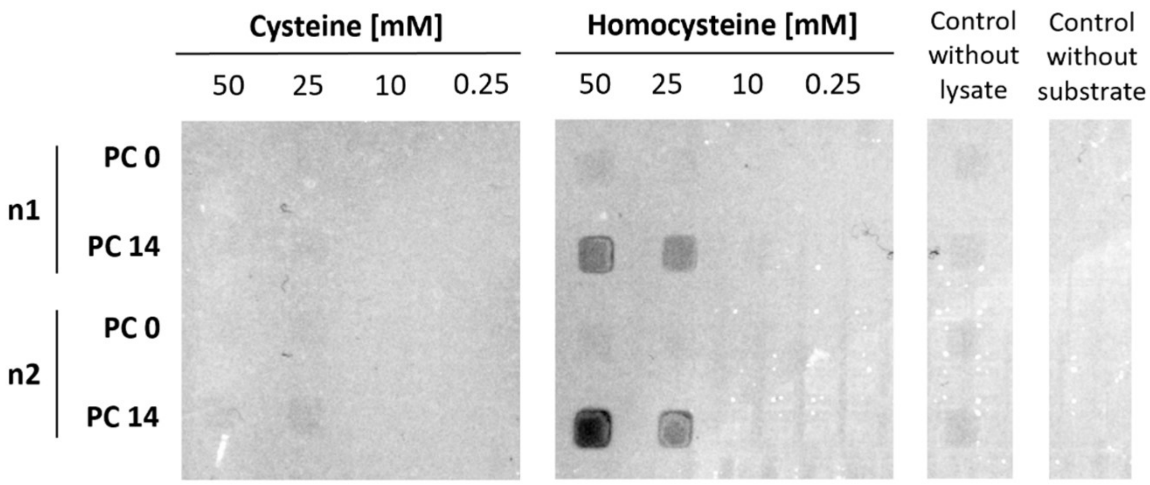

Figure S4. H_2S production in lysates from undifferentiated and terminally differentiated Caco-2 cells, trying different concentrations of cysteine and homocysteine.

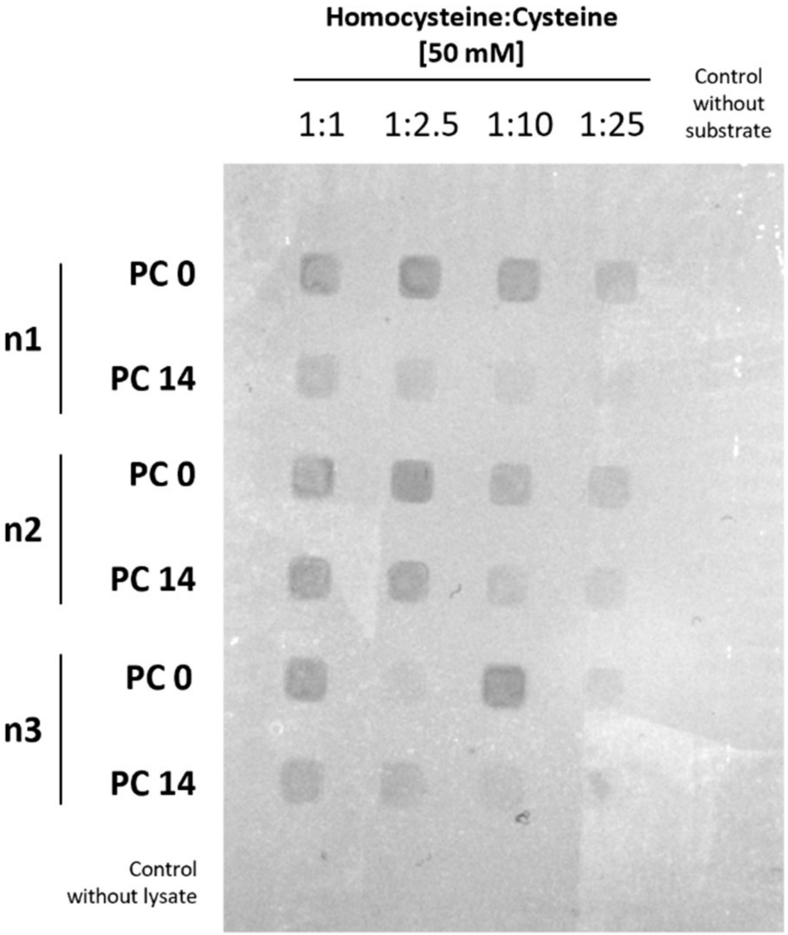

Figure S5. H_2S production in lysates from undifferentiated and terminally differentiated Caco-2 cells, trying different ratios of cysteine and homocysteine concentrations.
